# Synergistic activity of *Limosilactobacillus reuteri* KUB-AC5 and water-based plants against *Salmonella* challenge in a human in vitro gut model

**DOI:** 10.1038/s41598-024-53912-5

**Published:** 2024-02-27

**Authors:** Kevin Mok, Orranich Honwichit, Thanyakan Funnuam, Suvimol Charoensiddhi, Sunee Nitisinprasert, Dennis Sandris Nielsen, Massalin Nakphaichit

**Affiliations:** 1 Department of Biotechnology, Faculty of Agro-Industry, Kasetsart University, Bangkok, Thailand; 2 Specialized Research Unit: Probiotics and Prebiotics for Health, Faculty of Agro-Industry, Kasetsart University, Bangkok, Thailand; 3 Department of Food Science and Technology, Faculty of Agro‑Industry, Kasetsart University, Bangkok, Thailand; 4 Department of Food Science, University of Copenhagen, Frederiksberg, Denmark

**Keywords:** Metagenomics, Microbial ecology, Microbiome

## Abstract

A synbiotic is a combination of live microorganisms and specific substrates that are selectively utilized by host microorganisms, resulting in health benefits for the host. Previous studies have demonstrated the protective effects of *L. reuteri* KUB-AC5 against *Salmonella* infection in chicken and mouse models. The probiotic activity of *L. reuteri* KUB-AC5 in these hosts was influenced by nutritional supplements. Water-based plants contain significant amounts of carbohydrates, particularly dietary fiber and proteins, making them potential prebiotic substrates. In this study, four water-based plants (*Ulva rigida*, *Caulerpa lentillifera*, *Wolffia globosa*, and *Gracillaria fisheri*) were screened for their ability to support the growth of *L. reuteri* KUB-AC5. Under monoculture testing, *U. rigida* exhibited the highest capacity to support the growth of *L. reuteri* KUB-AC5 and the production of organic acids, including acetic acid, lactic acid, and propionic acid (*p* ≤ 0.05). In co-culture experiments, the synbiotic combination of *U. rigida* and *L. reuteri* KUB-AC5 demonstrated the potential to eliminate *Salmonella* Typhimurium DMST 48437 when inoculated at 10^4^ CFU/mL within 9 h. The synbiotic activities of *U. rigida* and *L. reuteri* KUB-AC5 were further investigated using an in vitro human gut model. Compared to the probiotic treatment, the synbiotic combination of *L. reuteri* KUB-AC5 and *U. rigida* showed significantly higher levels of *L. reuteri* KUB-AC5 (5.1 log copies/mL) and a reduction of *S*. Typhimurium by 0.8 log (CFU/ml) after 24 h (*p* ≤ 0.05). Synbiotic treatment also significantly promoted the production of short-chain fatty acids (SCFAs), including butyric acid, propionic acid, and acetic acid, compared to prebiotic and probiotic treatments alone (*p* ≤ 0.05). Furthermore, the synbiotic formulation modulated the in vitro simulated gut microbiome, enhancing putatively beneficial gut microbes, including lactobacilli, *Faecalibacterium*, and *Blautia*. Our findings demonstrated that *L*. *reuteri* KUB-AC5, in combination with *U*. *rigida*, exhibited synergistic activity, as indicated by increased viability, higher anti-pathogenicity toward *Salmonella*, and the ability to modulate the gut microbiome.

## Introduction

*Salmonella* infections cause significant problems, especially in developing countries, resulting in thousands of fatalities each year^1,2^. There is also a fear that the rising prevalence of multidrug-resistant *Salmonella* in the food supply chain will exacerbate this problem in the future^3,4^. Therefore, it is crucial to consider ways to prevent and minimize *Salmonella* infection in humans and livestock^5,6^. *Limosilactobacillus reuteri* KUB-AC5 has been shown to reduce salmonellosis in the poultry industry^7–9^ by producing antimicrobial substances that are effective against various *Salmonella* strains^10^*.* A previous study also reported that KUB-AC5 reduced *Salmonella* infection in mouse colitis models with attenuated gut inflammation in a cell density-dependent manner^5,8^.

A prebiotic is described as a substrate that is selectively utilized by host microorganisms to confer health benefits^11^. Water-based plants, particularly seaweed and duckweed, are rich sources of dietary fiber, proteins, polyunsaturated fatty acids, and minerals^12^. They have become attractive sources for commercial applications because of their fast growth rates and lack of arable land requirements compared to terrestrial plants^13^, with high dietary fiber content making them potential prebiotic candidates^14^. Several studies have shown that seaweed and duckweed are metabolizable by gut microbiota, thereby generating beneficial short-chain fatty acids (SCFAs)^12,15–17^.

A mixture of live microorganisms and a substrate that is selectively utilized by host microorganisms to promote host health is described as a synbiotic^18^. In recent years, the benefits of combining probiotics and prebiotics compared to their individual use have been increasingly recognized, with synbiotics demonstrating enhanced efficacy in various aspects of gut health. Studies have shown that synbiotics are more effective in reducing intestinal NH_4_^+^ levels, which is highly beneficial because it helps reduce the potential for gut dysbiosis. Moreover, lower levels of ammonium have been shown to create a conducive environment for the growth of butyric acid-producing bacteria^19^.

In an in vivo study involving mice with induced inflammatory bowel disease (IBD), the combination of *Bacillus coagulans* MTCC 5856 with sugarcane cane fiber exhibited a higher anti-inflammatory effect, reduced disease severity, and demonstrated superior modulation of metabolite and short-chain fatty acid (SCFA) profiles compared to probiotic or prebiotic treatment alone^20^.

A recent consensus by the International Scientific Association for Probiotics and Prebiotics (ISAPP)^18^ determined two main approaches for synbiotic formulation. The first is a complementary synbiotic with a formulation consisting of a recognized probiotic strain and a prebiotic substrate, where the probiotic and prebiotic do not necessarily have synergistic effects. The second is a synergistic synbiotic, where the substrate is designed to be selectively utilized by the probiotic; however, the prebiotic might also stimulate other beneficial members of the gastrointestinal microbiota^21^.

Addressing *Salmonella* infections has encountered challenges, including the emergence of drug-resistant strains and limitations in existing preventive strategies. Synbiotic represent a novel approach that harnesses the combined strength of probiotics and prebiotics. The choice of water-based plants as potential prebiotic substrates exhibits major advantages, with their attributes extending beyond mere high dietary fiber content^12^. These plants harbor an array of components and a diverse nutritional profile, including proteins, polyunsaturated fatty acids, and essential minerals. Moreover, with their fast growth rates and minimal arable land requirements, these plants provide a rich array of components that could foster a conducive environment for beneficial gut microorganisms.

In this study, we hypothesized that combining water-based plants with *L. reuteri* KUB-AC5 might enhance the overall functional capabilities, such as supporting the bacterial strain, stimulating specific groups of desirable gut microbes, and increasing the production of SCFAs. To prove or disprove this hypothesis, we investigated the efficacy of a synbiotic combination of *L. reuteri* KUB-AC5 and water-based plants during *Salmonella* challenge in an in vitro simulated human gut model. The gut model replicated the human gut’s environment and contained a human-like gut microbiome, enabling to explore the interaction between the human microbiome, synbiotics, and *Salmonella.*

## Results

### Nutrient composition of potential prebiotics from water-based plants

To formulate a functional synbiotic, the substrate must provide sufficient nutrients to support the growth of the co-administered bacteria. Here, we examined the fat, protein, ash, carbohydrate, and dietary fiber contents of water-based plants using proximate analysis (Table 1). More than 80% of the dry matter from all samples was carbohydrate and protein, except *C. lentilifera,* where ash and carbohydrate were the major compounds. *Gracillaria fisheri* had the highest carbohydrate content among the four tested water plants, followed by *C. lentilifera* and *U. rigida*, with the highest protein content found in *W. globosa* and *U. rigida* (Table 1).

**Table 1:** Proximate composition percentages of potential prebiotics from water-based plants (g/100 g dry weight).

Analysis	*Ulva rigida*	*Caulerpa lentillifera*	*Wolffia globosa*	*Gracillaria fisheri*
Fat (Mojonnier extraction)	2.31	2.42	3.00	1.60
Protein (N*6.25)	32.34	7.89	36.36	15.95
Ash	19.06	40.07	19.40	11.54
Carbohydrates	46.29	49.63	41.24	70.91
Dietary fiber				
Soluble	23.63	6.15	3.50	42.21
Insoluble	19.65	16.08	15.08	22.34
Total	43.28	22.23	18.58	64.55
Carbohydrate: Protein	1.4	6.3	1.1	4.5

Among the four water-based plants examined in this study, *C. lentillifera* and *G. fisherii* exhibited the highest carbohydrate and protein ratios, while *U. rigida* and *W. globosa* displayed balanced carbohydrate and protein ratios approaching a 1:1 proportion. These findings highlight the varying nutritional composition of the four plant species and provide insights into their suitability as substrates for microbial metabolism and growth (Table 1).

### Survival of *Limosilactobacillus reuteri* KUB-AC5 during in vitro simulated passage through the upper gastrointestinal tract

**Figure 1:**
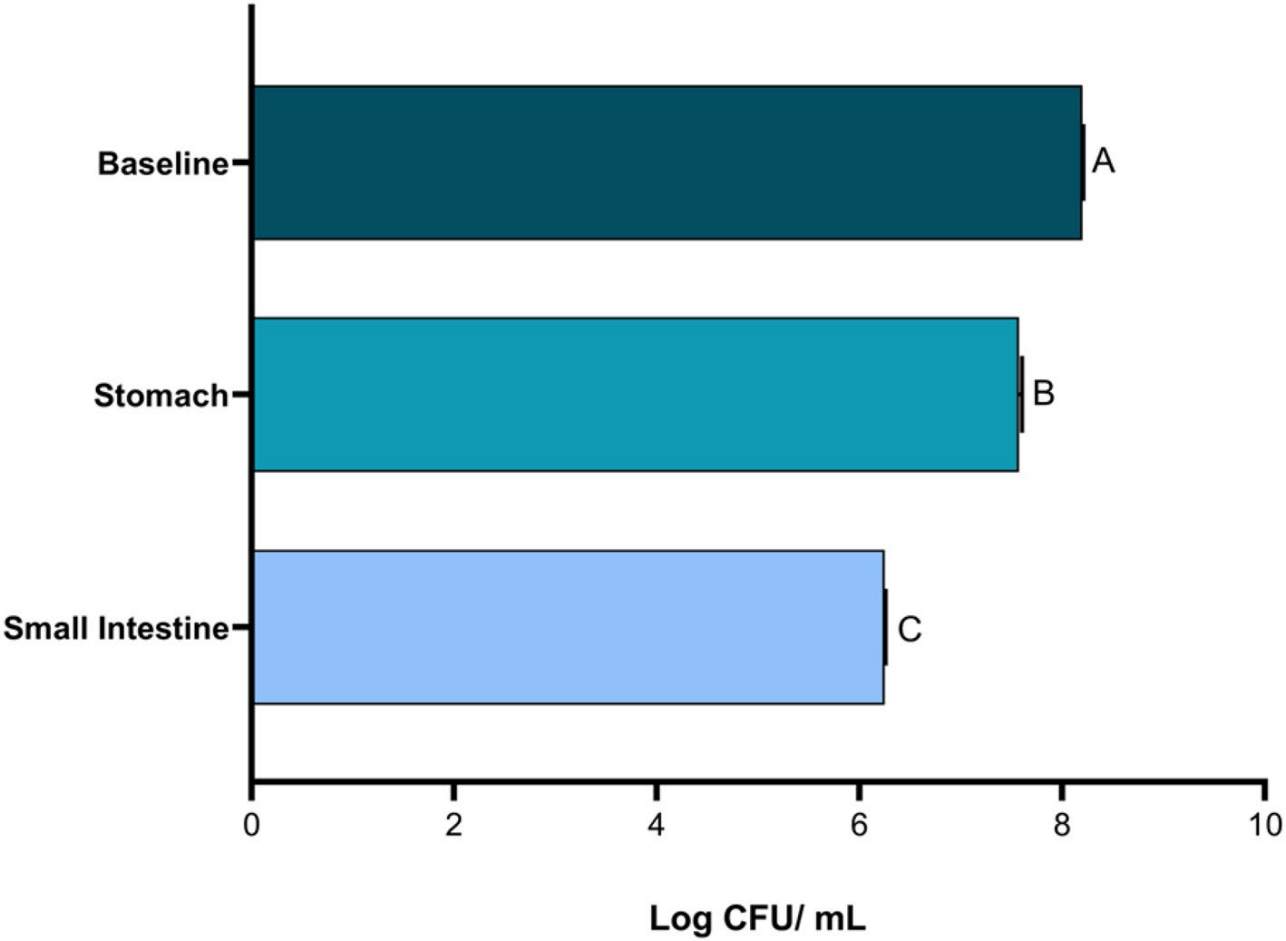
Survival of *Limosilactobacillus reuteri* KUB-AC5 during incubation at stomach-like and small intestine-like conditions for 2.5 and 4 h, respectively. Standard deviation indicated. Different letters (A, B, C) denote significant differences in viability (*p* < 0.05).

The ability of *L. reute*ri KUB-AC5 to survive gastric conditions was tested, and a modest 0.7 log CFU/mL reduction after 2.5 h in simulated gastric juice was found, with a further reduction of 2 log CFU/mL observed after 4 h of exposure to artificial small intestinal fluid (Fig. 1).

### Determination of the ability of water-based plants to support the growth of *Limosilactobacillus reuteri* KUB-AC5

**Figure 2:**
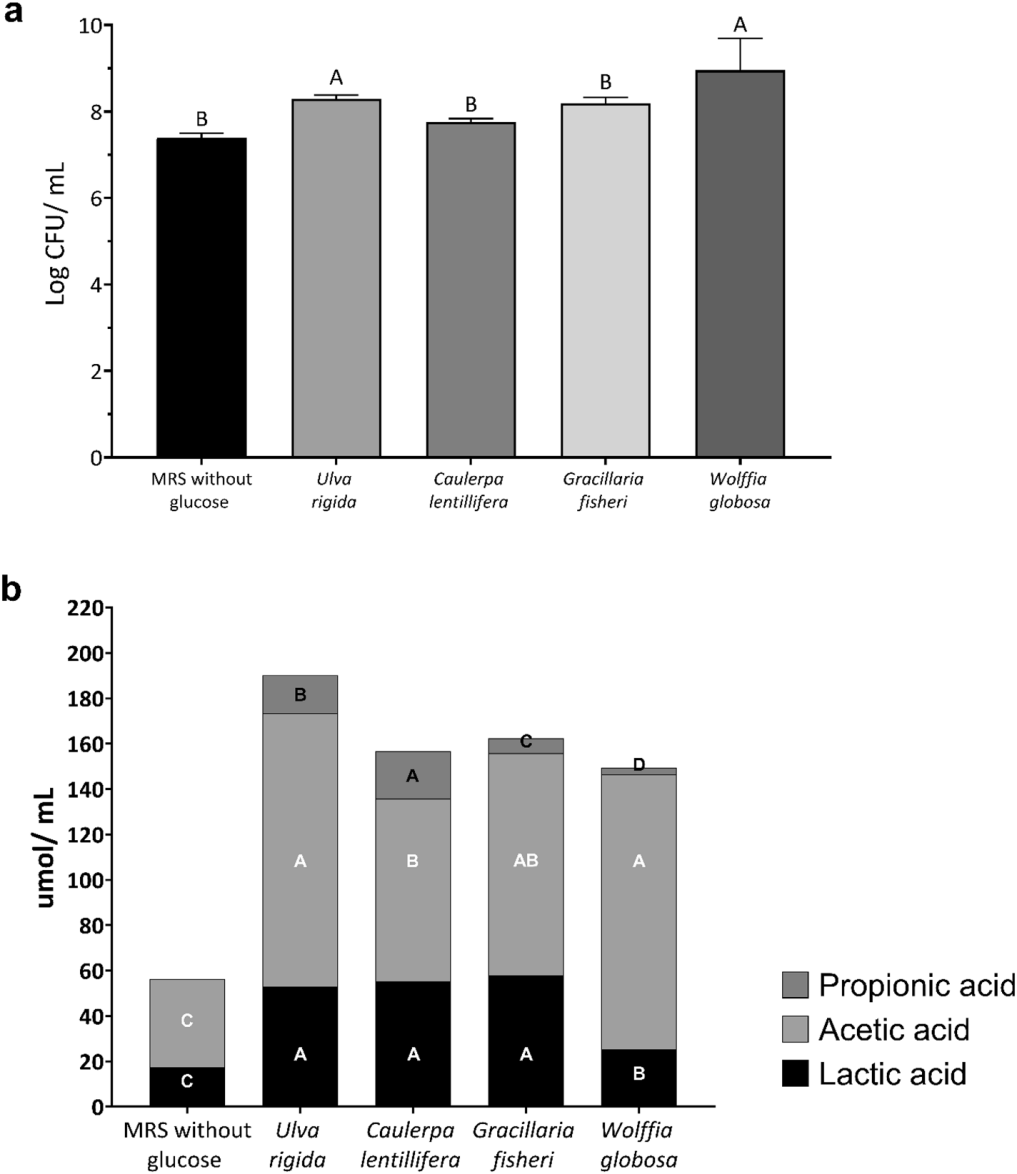
Effect of water-based plants powder supplementation on growth (**a**) and organic acid production (**b**) of *Limosilactobacillus reuteri* KUB-AC5 grown at 37 ℃ without agitation. Standard deviation was indicated. Bars with different letters in each acid are significantly different (*p* < 0.05).

The ability of *L. reuteri* KUB-AC5 to utilize water-based plant powders as carbon sources was investigated by substituting glucose in MRS broth with four different water-based plant powders (4% w/v) (Fig. 2a). Among the water-based plant powders tested, only *U. rigida* and *W. globosa* demonstrated significant growth-promoting effects compared to the negative control (MRS without added carbohydrate), reaching 8.29 and 8.96 log CFU/mL, respectively. The production of propionic acid, acetic acid, and lactic acid was also analyzed, and the results indicated that the levels of these organic acids produced by *L. reuteri* KUB-AC5 in the presence of *U. rigida* were the highest compared to those in other water-based plants (Fig. 2b). Therefore, *U. rigida* was selected to form a synbiotic with *L. reuteri* KUB-AC5.

### Anti-Salmonella activity of *Limosilactobacillus reuteri* KUB-AC5 grown in the presence of *Ulva rigida*

**Figure 3:**
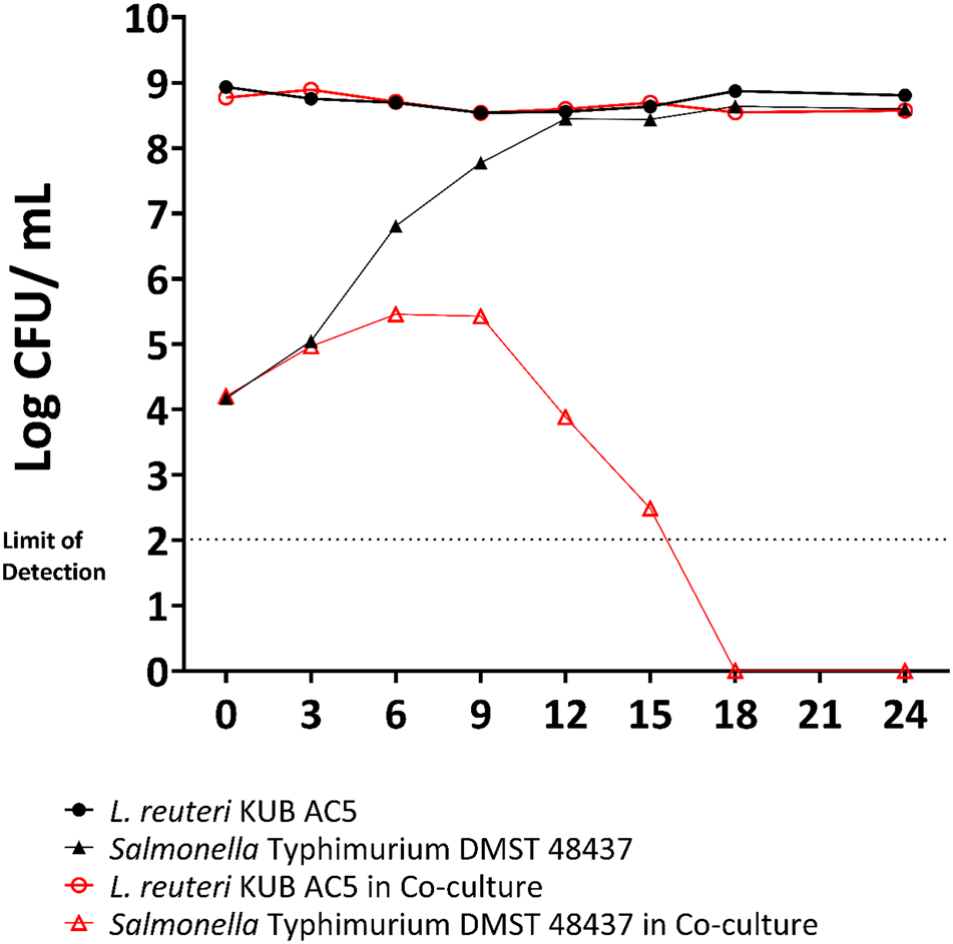
Growth of *Limosilactobacillus reuteri* KUB-AC5 (red circle) and *Salmonella* Typhimurium DMST 48437 (red triangle) co-cultured in gut basal medium with *Ulva rigida* supplement. Single cultures of *L. reuteri* KUB-AC5 (black circle) and *S.* Typhimurium DMST 48437 (black triangle) in gut basal medium with *Ulva rigida* supplement were used as controls.

The anti-*Salmonella* activity of *L. reuteri* KUB-AC5 with *U. rigida* was investigated by co-cultivation with *S.* Typhimurium DMST 48437. The growth of *S.* Typhimurium DMST 48437 increased from 4 log CFU/mL to 5 log CFU/mL after 9 h and then suddenly decreased from 5 log CFU/mL to below the detection limit (10^2^ CFU/mL) after 18 h. In contrast, the growth of *S.* Typhimurium DMST 48437 grown as a single culture increased from 4 to 8 log CFU/mL after 12 h. The growth curves of *L. reuteri* KUB-AC5 in both single and co-cultivation were similar and stable at 9 log CFU/mL for 24 h. This indicates that the combination of *L. reuteri* KUB-AC5 with *U. rigida* showed enhanced inhibitory activity against *S.* Typhimurium DMST 48437 (Fig. 3) relative to the individual constituents.

### Effect of *Limosilactobacillus reuteri* KUB-AC5 and *Ulva rigida* synbiotic on an in vitro simulated human colon microbiome exposed to a *Salmonella* challenge

**Figure 4:**
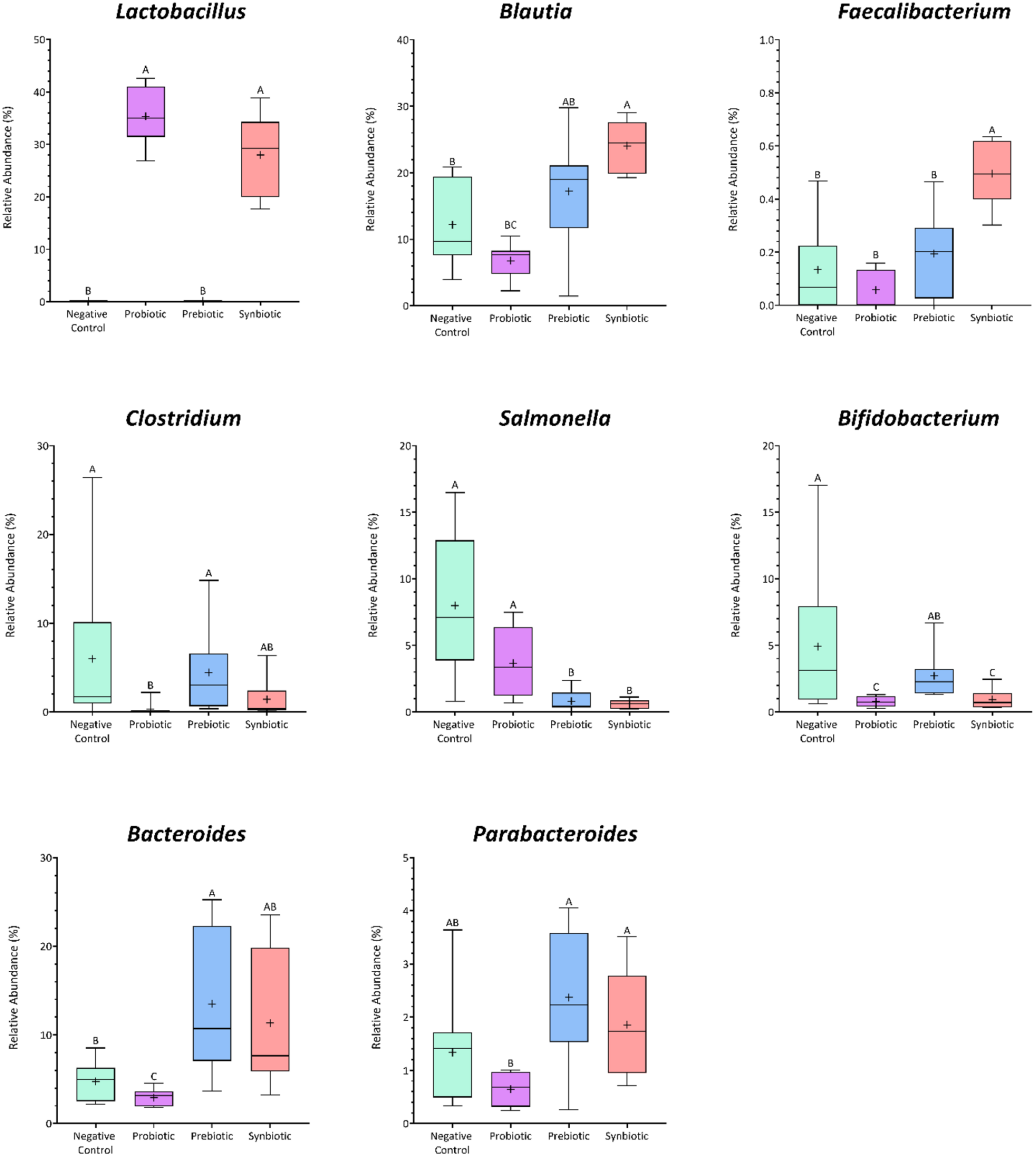
Relative abundance of significantly differently abundant key phylotypes from in vitro simulated human colon passage at genus level based on LEfSe analysis. (*p* < 0.01 and LDA score > 3).

As shown in Fig. S1, the richness and diversity indices of the in vitro simulated colon microbiome did not differ between the negative control and the prebiotic group, while the probiotic and synbiotic fermentations led to significantly lower values (*p* ≤ 0.05). The linear discriminant analysis (LDA) effect size (LEfSe) approach was applied to identify differences in the gut microbiota composition between treatments at 24 h. Distinctive phylotypes were found to be linked to the treatment (Fig. 4). The gut microbiome in each treatment group exhibited a different pattern. *Salmonella*, *Clostridium*, and *Bifidobacterium* constituted a large fraction of the bacteria present in the negative control, and the probiotic treatment showed an abundance of lactobacilli, whereas *Bacteroides* and *Parabacteroides* were significantly more abundant in the prebiotic and synbiotic treatments. *Faecalibacterium* and *Blautia* were also highest in the synbiotic treatments (Fig. 4).

### Anti-Salmonella activity of *Limosilactobacillus reuteri* KUB-AC5 and *Ulva rigida* in an in vitro simulated human gut model

**Figure 5:**
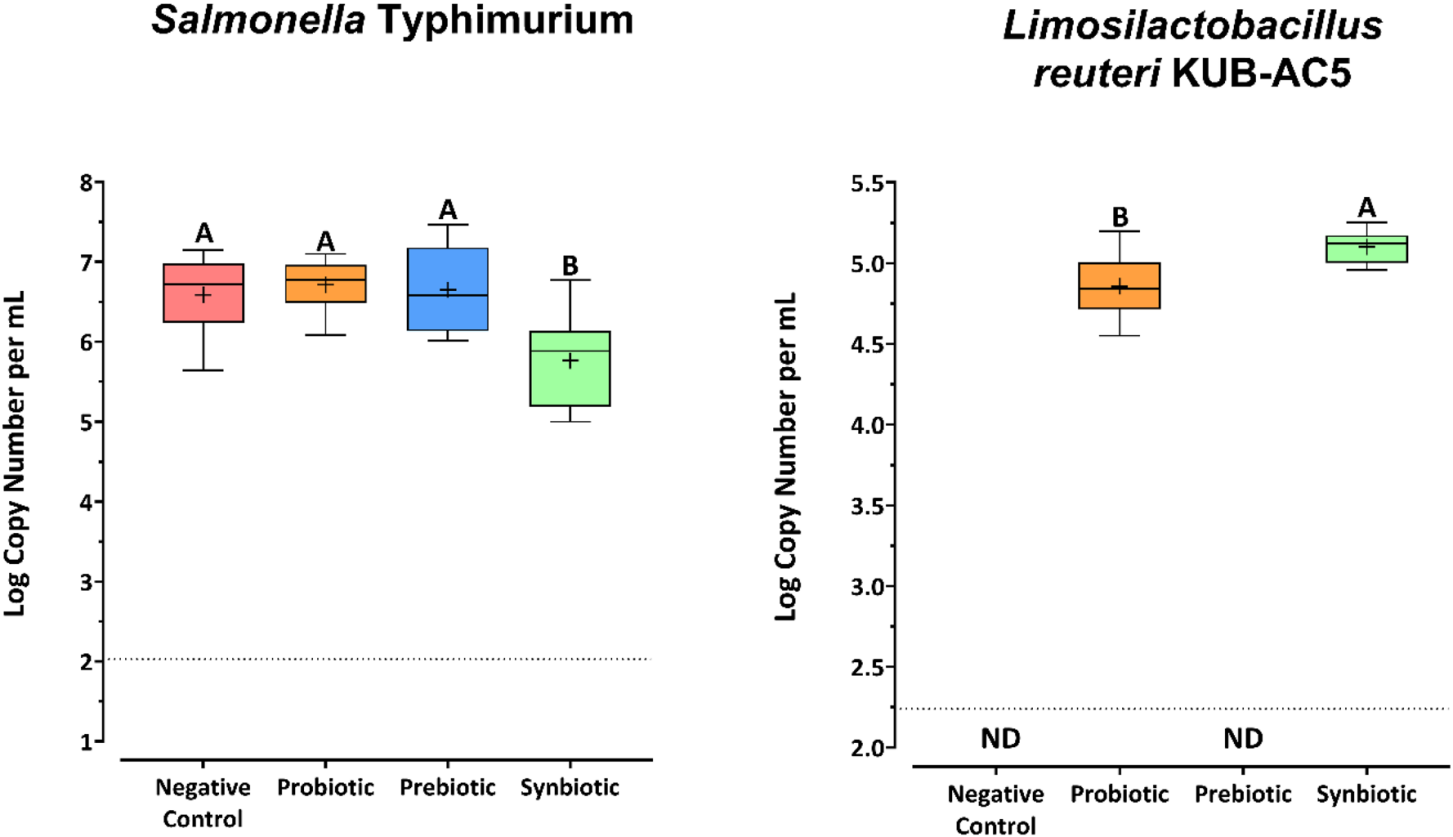
Absolute quantification of *Salmonella* Typhimurium and *Limosilactobacillus reuteri* KUB-AC5 after 24 h of in vitro simulated colon passage. The detection limit for each primer is shown as a dotted line. Different letters (A, B) indicate significant differences between the four treatments (*p* < 0.05).

The *Salmonella* inhibitory activity of *L. reuteri* KUB-AC5 and *U. rigida* was investigated during in vitro simulation of the human colon model. The level of *S.* Typhimurium was quantified using quantitative real-time PCR (Fig. 5). Interestingly, *S.* Typhimurium constituted between 6.59 and 6.71 log (copies/mL) in the negative control, probiotic, and prebiotic treatments. This was significantly higher than the synbiotic treatment, resulting in 5.76 log (copies/mL) (*p* ≤ 0.05). This indicated that only the combination of *L. reuteri* KUB-AC5 and *U. rigida* inhibited *Salmonella.*

The level of *L. reuteri* KUB-AC5 in the human gut model was quantified using species-specific primers and quantitative real-time PCR. *L. reuteri* KUB-AC5 was detected only in the probiotic and synbiotic treatments, suggesting that donors in the gut microbiome did not contain *L. reuteri* KUB-AC5. The concentration of *L. reuteri* KUB-AC5 in the synbiotic treatment was significantly higher than that in the probiotic treatment (*p* ≤ 0.05), suggesting that *L. reuteri* KUB-AC5 utilized *U. rigida* during in vitro simulated colon passage.

### The effect of *Limosilactobacillus reuteri* KUB-AC5 and *Ulva rigida* on SCFA production in an in vitro simulated colon model

**Figure 6:**
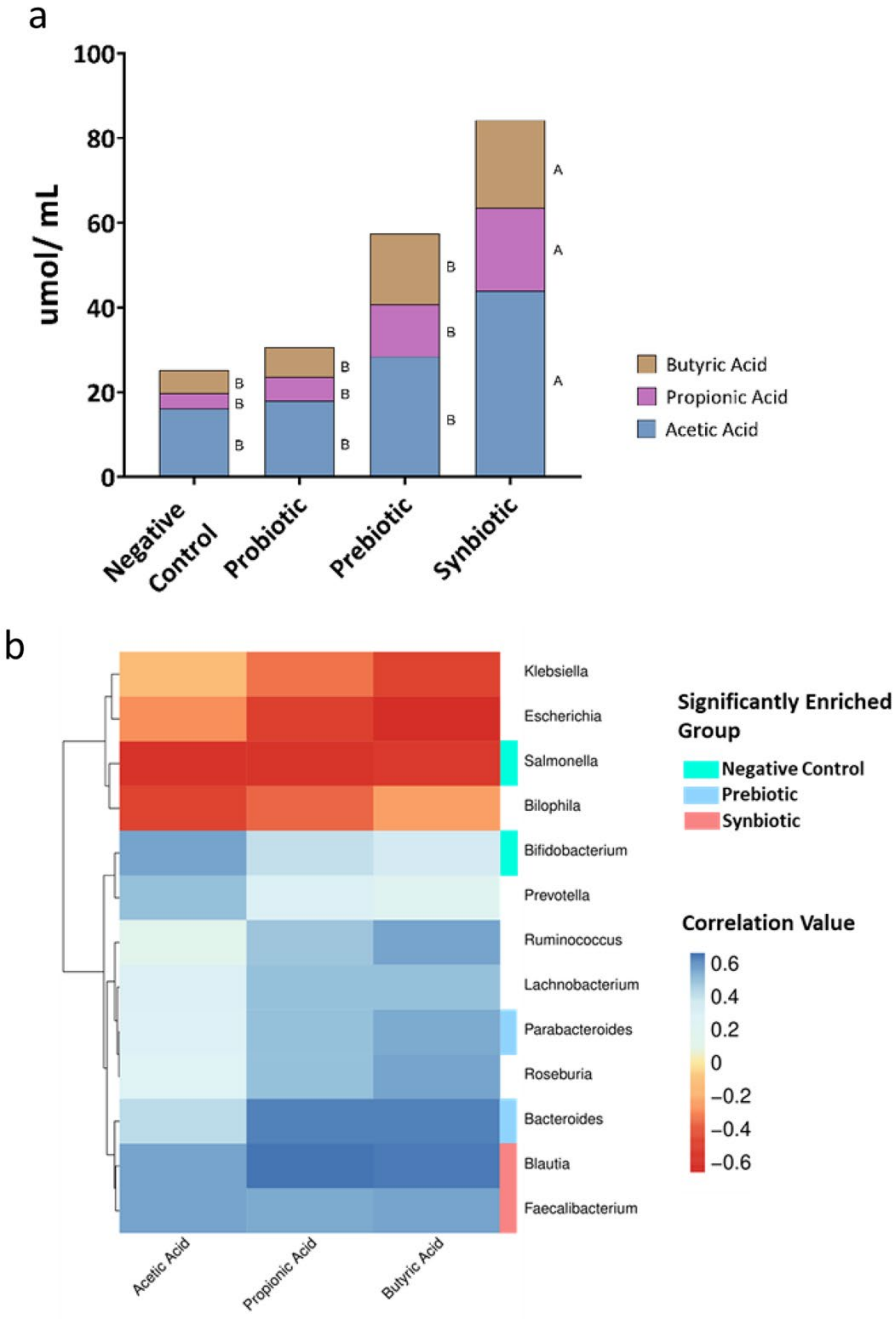
(**a**) Short-chain fatty acid (SCFA) profiles after 24 h of in vitro simulated colon passage. Different letters (A, B) beside the bar graph indicate significantly different concentrations of the SCFA in question (*p* < 0.05). (**b**) Pearson correlation analysis between SCFA and bacterial taxa (genus level). Only correlations with *p* < 0.05 are shown.

Acetic, propionic, and butyric acids were identified in all treatments. The level of SCFAs in the synbiotic treatment was the highest, followed by the prebiotic, probiotic, and negative control (*p* ≤ 0.05). Interestingly, SCFA production in the synbiotic treatment was 1.6-fold higher than that in the prebiotic treatment, possibly because the synbiotic treatment enriched some bacterial taxa that could produce organic acids (Fig. 6a).

Pearson’s correlation analysis (Pearson) was used to identify bacterial taxa that correlated well with organic acid secretion. Overall, SCFA production was positively correlated with bacteria enriched in the prebiotic and synbiotic groups but inversely correlated with abundance in the negative control treatment (Fig. 6b). The strongest positive correlation in the synbiotic treatment was observed for *Blautia* with propionic and butyric acids and *Faecalibacterium* with butyric acid. A strong negative correlation was also observed between *Enterobacteriaceae* family members (*Escherichia*, *Salmonella*, and *Klebsiella*) and overall SCFA production.

## Discussion

Prebiotics play a crucial role in promoting the growth and colonization of probiotic bacteria in the gut. By providing a favorable environment, synbiotics increase the chances of probiotics surviving and thriving in the gastrointestinal tract, which can lead to greater health benefits. To enhance the activity of *L. reuteri* KUB-AC5 in the human gut environment, four water-based plants were screened. *Ulva rigida* and *W. globosa* showed the best growth-promoting effects, while in in vitro co-culture, *U. rigida* also demonstrated a higher capacity for supporting the production of organic acids compared to other water-based plants (Fig. 2). *Limosilactobacillus reuteri* KUB-AC5 displayed robust growth in *U. rigida* and *W. globosa*, which had similar carbohydrate-to-protein ratios of approximately 1:1. In contrast, the other two water-based plants had carbohydrate–protein ratios ranging from 4 to 6. Interestingly, the 1:1 carbohydrate-to-protein ratio is comparable to that of MRS media, which is commonly used for culturing *Lactobacillus* spp.^22^. Furthermore, a recent study reported that *Lactobacillus* spp. utilize *U. rigida* and enhance the production of SCFAs^23^.

Our previous report showed that *L. reuteri* KUB-AC5 produced antimicrobial substances including short peptide and lactic acid against *Salmonella*. However, the inhibitory activity varies depending on the gut host environment^5,8,24^. The variation in *L. reuteri* KUB-AC5 against *Salmonella* in diverse gut environments emphasizes the importance of optimizing working conditions through synbiotic combinations. The addition of prebiotics can significantly contribute to lowering pH levels by offering a readily accessible substrate. This addition also might help to induce the production of SCFA, thus increasing the antimicrobial capabilities of *L. reuteri*. Therefore, a synbiotic combination of *L. reuteri* KUB-AC5 and *U. rigida* was formulated to evaluate its effectiveness in modulating gut microbiota composition and inhibiting the growth of *Salmonella* using an in vitro simulated human gut model. Our results showed that *U. rigida* was selectively utilized by *L. reuteri* KUB-AC5, leading to significantly higher levels of *L. reuteri* KUB-AC5 than probiotic treatment alone.

Elevated growth of *L. reuteri* KUB-AC5 in synbiotic treatment led to the inhibition of *Salmonella* during in vitro simulated colon passage. However, the inhibitory activity of *L. reuteri* KUB-AC5 in this model was lower than that of *L. reuteri* KUB-AC5 grown under co-culture conditions. *Salmonella* challenge disrupted the microbiome balance in the human gut model by promoting the proliferation of pathogenic bacteria within the *Enterobacteriaceae* family. Notably, the increased abundance of *Enterobacteriaceae* and *Rikenellaceae* was negatively correlated with the production of SCFAs, such as acetic acid, propionic acid, and butyric acid. Low levels of SCFAs in the gut have been linked to an increased risk of colorectal cancer, ulcerative colitis, and Crohn's disease^25–27^. Conversely, SCFAs enhance gut health by maintaining intestinal barrier integrity and protecting against inflammation^28^.

Both probiotic and synbiotic treatments affect the composition of the gut microbiota by increasing the abundance of *Lactobacillaceae* family members. Synbiotic treatment also influenced other beneficial gut microbes, including *Faecalibacterium* and *Blautia*, while prebiotic treatment encouraged the growth of *Bacteroides* and *Parabacteroidetes*. Pearson’s correlation analysis revealed a strong association between SCFAs and *Faecalibacterium* and *Blautia*, which were enriched in the synbiotic treatment. These findings suggest that synbiotic interventions might assist in providing favorable conditions for modulating the gut microbiome and contribute to the suppression of *Salmonella* in the gut.

Previous research has shown that SCFA depletion is associated with *Salmonella* infections in pigs ^29^. Consistent with this finding, reduced fecal SCFA levels in children with salmonellosis have also been observed^30^. Both studies used probiotics to ameliorate infection and recorded increased levels of SCFAs. Higher SCFA levels were also linked to milder symptoms in children with salmonellosis, and probiotic administration demonstrated protective properties against future infection in piglets. These results indicate that SCFA-producing probiotics may play a prominent role in the treatment of *Salmonella* infections.

## Materials and methods

### Bacterial cultures and potential prebiotic powder origin

*Limosilactobacillus reuteri* KUB-AC5 was supplied by the Specialized Research Unit of Probiotics and Prebiotics for Health, Department of Biotechnology, Kasetsart University, Thailand, and *Salmonella enterica* serovar Typhimurium was provided by the Research and Development Center, Betagro Agro-Group Public Co., Ltd., Thailand. *Limosilactobacillus reuteri* KUB-AC5 was maintained on MRS agar (Difco Laboratories, Detroit, MI, USA) supplemented with 0.5% CaCO_3,_ whereas *S.* Typhimurium DMST 48437 was maintained on nutrient agar (Oxoid, Basingstoke, UK). All isolates were preserved in nutrient broth or MRS broth supplemented with 40% glycerol and stored at -80 °C.

Green seaweed (*U. rigida* and *C. lentillifera*) and red seaweed (*G. fisheri*) were collected from a seaweed farm in southern Thailand between May and June 2021, with support from the Phetchaburi Coastal Fisheries Research and Development Center, Department of Fisheries, Ministry of Agriculture and Cooperatives. Duckweed (*W. globosa*) was provided by the Advanced GreenFarm Co., Ltd. (Nakhon Pathom, Thailand). After harvesting, the seaweeds were washed with tap water to remove any visible surface contaminants and dried in an oven at 60 °C for 8 h to obtain a moisture content of approximately 10%. The seaweeds were then finely ground using a hammer mill and sieved through a 0.2 mm mesh. All seaweed and duckweed were collected and prepared simultaneously to obtain consistent samples for the entire study. The ground sample powders were stored at room temperature in a desiccator until further use.

### Proximate analysis of water-based plants

The chemical composition of the ground samples was determined by proximate analysis, following the methods described by the Association of Official Analytical Chemists^31^. Moisture, protein, ash, crude fiber, and fat contents were analyzed using a hot air oven, N-Kjeldahl × 6.25, ignition at 550 °C, Fibertec™, and Soxhlet extraction, respectively. The carbohydrate content was calculated using the available carbohydrate by the difference (CHOAVDF) method. The total dietary fiber content, including soluble and insoluble fiber, was also analyzed according to the AOAC^32^.

### Preparation of bacterial cultures

*Limosilactobacillus reuteri* KUB-AC5 and *S*. Typhimurium inocula were cultivated in MRS broth (Difco Laboratories, Detroit, MI, USA) and nutrient broth (Oxoid, Basingstoke, UK), respectively. *Salmonella* Typhimurium was grown with agitation (120 rpm) for 18 h, whereas *L. reuteri* was cultured under static conditions for 15 h. Both strains were incubated at 37 °C. After centrifugation at 13,000×*g* for 3 min at 4 °C, the supernatant was discarded, and the pelleted cells were resuspended in an equal volume of phosphate buffer pH 7 before each experiment.

### Screening of water-based plants as substrates for the growth of *Limosilactobacillus reuteri* KUB-AC5

Modified MRS without glucose^33^ supplemented with 4% w/v water-based plant extract in sterile 50 mL Duran bottles and 2% v/v *L. reuteri* KUB-AC5 culture (adjusted to 10^8^ CFU/mL) was used to test the ability of the extracts to support *L. reuteri* KUB-AC5 growth during incubation at 37 °C for 15 h without agitation. Growth was determined by plating on MRS agar (Difco Laboratories, Detroit, MI, USA) and is presented as log CFU/mL. All experiments were performed in duplicate. For SCFA analysis, the culture broth was centrifuged twice at 13,000×*g* for 5 min, and the supernatants were stored at − 20 °C until analysis by high-performance liquid chromatography.

### Survival of *Limosilactobacillus reuteri* KUB-AC5 under in vitro simulated upper gastrointestinal tract conditions

To examine the ability of *L. reuteri* KUB-AC5 to survive the harsh conditions of the human gastrointestinal tract, the bacterium was exposed to in vitro simulated stomach and small intestine conditions. For the stomach phase, artificial gastric fluids consisting of a pH 2.5 solution of pepsin (Oxoid, Basingstoke, UK) (2000 UA/mL) in phosphate buffer were used, while for the small intestine phase, artificial intestinal fluids consisting of a pH 8 solution of pancreatin (200 UA/mL) and 0.3% bile salt were used ^34–36^. The number of viable cells was assessed sequentially in each phase of the artificial gastrointestinal tract (baseline, stomach, and small intestine).

The artificial stomach condition was initiated by mixing *L. reuteri* KUB-AC5 suspensions (10^9^ CFU/mL) with 9 mL of artificial gastric fluid. The mixture was then incubated at 37 °C with homogenization every 20 min for 2.5 h. To simulate passage to the small intestine, the artificial gastric fluid was removed by centrifugation, and the pelleted cells were resuspended in 10 mL of artificial intestinal fluid. The mixture was incubated at 37 °C for 4 h, with homogenization every 40 min. After each of the two stages, the artificial gastrointestinal fluids were removed by centrifugation, and the cells were resuspended and serially diluted with the maximum recovery diluent (Oxoid, Basingstoke, UK) before plating on MRS agar (Difco Laboratories, Detroit, MI, USA).

### Co‑cultivation of *Limosilactobacillus reuteri* KUB-AC5 and *Salmonella Typhimurium* supplemented with *Ulva rigida* powder

The effect of *U. rigida* supplementation on the ability of *L. reuteri* KUB-AC5 to inhibit the growth of *S*. Typhimurium was investigated in co-culture using gut basal medium (0.2% w/v peptone, 0.2% w/v yeast extract, 0.01% w/v NaCl, 0.004% w/v K_2_HPO_4_, 0.004% w/v KH_2_PO_4_, 0.001% w/v MgSO_4_, 0.001% CaCl_2_, 0.2% w/v NaHCO_3_, 0.2% v/v Tween 80, 0.5% w/v bile salt, 0.001% v/v Antifoam SE15, and 0.001% w/v resazurin) with 4% w/v *U. rigida* powder.

For the assay, *L. reuteri* KUB-AC5 and *Salmonella* Typhimurium were seeded into gut basal broth supplemented with *U. rigida* at final concentrations of 10^9^ and 10^4^ CFU/mL, respectively. The adjustment process was performed by measuring the OD_600_ of each strain with a spectrophotometer and was confirmed by plating each inoculum onto its respective medium. Monocultures of each strain in the same medium were used as controls. All tubes were then incubated under static conditions at 37 °C for 24 h, followed by bacterial enumeration in selective media, MRS (Difco Laboratories, Detroit, MI, USA), and nutrient agar (Oxoid, Basingstoke, UK) for *L. reuteri* KUB-AC5 and *S.* Typhimurium DMST 48437, respectively. The results were reported as log CFU/mL. Each co-culture was performed in duplicate.

### Inhibition of *Salmonella* Typhimurium by *Limosilactobacillus reuteri* KUB-AC5 during in vitro simulated colon passage

The ability of *L. reuteri* KUB-AC5 to inhibit *Salmonella* Typhimurium during in vitro simulated colon passage with and without *U. rigida* supplementation was investigated using the CoMiniGut system, with experimental conditions designed to simulate colon transit for 24 h, as previously described ^37^. Fecal inocula were collected from three healthy adults who had not received antibiotic treatment or probiotics during the preceding 3 months. Informed consent from the volunteers were obtained before sample collection and the process was conducted according to the Helsinki Declaration. All protocol involving fecal sample collection from volunteers were approved by the Ethical Committee of the Capital Region of Denmark registration number H-20028549. The fecal slurries were individually homogenized in a 1:1 ratio with 1 M PBS and 20% glycerol (v/v) for 2 × 60 s using a Stomacher (Stomacher 400; Seward, Worthing, UK) at normal speed. Fecal slurries were then aliquoted and stored at − 80 °C until further use. Fecal slurry stocks were thawed and diluted with 0.1 M PBS pH 5.6 (1:4) on the day of the experiment.

Fecal batch fermentations were separated into four treatments: negative control, probiotic (*L. reuteri* KUB-AC5 at 8 log CFU/mL)), prebiotic (4% w/v of *U. rigida*), and synbiotic (*L. reuteri* KUB-AC5 at 8 log CFU/mL and 4% w/v of *U. rigida*). All treatments were spiked with *S. Typhimurium* DMST 48437 at 4 log CFU/mL. Each treatment was fermented in triplicate in a random order to avoid potential run effects. Samples were collected at 0 and 24 h for microbiome determination and metabolite analysis.

### Bacterial DNA extraction

Bacterial DNA was extracted according to the DNeasy PowerSoil Pro Kit protocol (Qiagen) from CoMiniGut fermentates at 0 h and 24 h of fermentation, with the cell pellet obtained from 125 to 200 µL of fermentation by centrifugation at 13,000×*g* for 10 min. The FastPrep bead-beating step was performed in two cycles of 30 s each at a speed of 2000 rpm in a FastPrep-24™ homogenizer (MP). DNA quantity and quality were measured using a Qubit^TM^4 Fluorometer (Thermo Scientific, Waltham, MA, USA).

### Microbiota profile analysis

The fecal microbiota composition in the gut model was determined using MinION (Oxford Nanopore Technologies, Oxford, UK), with PCR and library preparation of the V1–V8 hypervariable region of the 16S rRNA gene, as described previously^38^.

Data generated by MinION were collected using MinKnow software v19.06.8 (https://nanoporetech.com). The Guppy v3.2.2 base-calling toolkit was used to base raw fast5 to fastq (https://nanoporetech.com). Porechop v0.2.2 was used for adapter trimming and sample demultiplexing (https://github.com/rrwick/Porechop). Sequences containing quality scores (fastq files) were quality corrected using NanoFilt (q ≥ 10; read length > 1 kb). Taxonomy assignment of quality-corrected reads against the Greengenes (13.8) database was conducted using the uclust method implemented in parallel_assign_taxonomy_uclust.py (QIIME v1.9.1). The uclust settings were tuned to mock communities (similarity 0.8; min_consensus_fraction 0.51), assuring annotations to the lowest taxonomic level with no false positive annotations. The settings allowed the program to treat individual amplicon sequence variants as individual “seeds”. Reads classified at the minimum phylum level were subjected to further analysis. Alpha diversity matrices (Chao1 richness, Pielou’s evenness, and Shannon index) and Bray‒Curtis beta diversity were calculated based on the amplicon sequence variance (ASVs) table using USEARCH v11.0.667^39^.

### Quantification of *Limosilactobacillus reuteri* KUB-AC5 and *Salmonella* Typhimurium by quantitative PCR (qPCR)

*Limosilactobacillus reuteri* KUB-AC5 and *S.* Typhimurium were quantified by qPCR using the LightCycler480 platform (Roche, Basel, Switzerland). An oligonucleotide primer for *L. reuteri* KUB-AC5 was developed in this study based on the protein sequence of the antimicrobial peptide from *L. reuteri* KUB-AC5, which has a molecular weight of active AMP of 4.7 kDa as determined by MALDI-TOF mass spectrometry^10,24^. The protein sequence was converted to a DNA sequence and checked for specificity using the NCBI BLAST database (http://www.ncbi.nlm.nih.gov/BLAST, accessed on 23rd October 2019). Primer quality was verified using the PrimerQuest™ tool (IDT, Coralville, IA, USA). The unique putative primers of *L. reuteri* KUB-AC5 (AC-1792F: 5′-CGAAAATGGGAGTAATTAACTATGG-3′ and AC-1792R: 5′-ATTTCCTGCAGCTAAACTTCCA-3′) were validated for specificity against several Lactobacillus species and *Escherichia coli.* To obtain absolute quantification of *S*. Typhimurium, the STM4497 primer set was used, including STM4497F:5′-AACAACGGCTCCGGTAATGA and STM4497R3:5′-TGACAAACTCTTGATTCTGA^40^. The reaction mixture contained 10 μL of SsoAdvanced Universal SYBR Green Supermix (Bio-Rad, Hercules, CA, USA), 0.5 μL of 10 pmol/μL of the forward and reverse primers, 2 μL of DNA template (30–50 ng), and nuclease-free water added to obtain a final volume of 20 μL. The amplification conditions were as follows: initial denaturation at 95 °C for 5 min, 30 cycles of amplification with denaturation at 95 °C for 1 min, annealing at the primer specified temperature (56 °C for AC5-1792 and 53 °C for STM4497) for 15 s, extension at 72 °C for 25 s, and a final extension at 72 °C for 5 min. To confirm the specific amplification of the target DNA, a dissociation curve was created using a denaturation step at 95 °C for 5 s, decreased to 65 °C for 1 min, and continuously increased from 65 to 97 °C, with signal measurement every 12 s. Specific sizes of the PCR products were determined by gel electrophoresis.

Standard curves were constructed using specific primers to amplify plasmids containing DNA from *L. reuteri* KUB-AC5 and *S.* Typhimurium DMST 48437. A standard plasmid was constructed following a previously described method^41^. Ten-fold serial dilutions of the plasmid, ranging from 10^1^ to 10^8^ copies/μL, were used to create a standard curve.

### Metabolite analysis of fecal fermentation broths

Fatty acids in in vitro colon-simulated fecal samples were quantified by high-performance liquid chromatography (Waters, Milford, MA, USA) using a UV detector at a wavelength of 210 nm. The sample supernatant was passed through a 0.22 µM filter (Vertical, Santa Clara, CA, USA) and mixed with 0.2% v/v tartaric acid (2:1). For SCFA separation, an Aminex HPX-87H column (300 × 7.8 mm) (Bio-Rad, Hercules, CA, USA) served as the static phase, with 8 mM sulfuric acid as the mobile phase. The flow rate was set at 0.6 mL/minute, and the injector and detector temperatures were maintained at 55 °C. Standard curves were constructed by injecting solutions of lactic acid, acetic acid, propionic acid, and butyric acid at concentrations between 0.9375 and 83 mmol/mL.

### Statistical analysis

All data used for statistical purposes were checked using the Shapiro‒Wilk normality test in GraphPad Prism version 8.4.3. ANOVA was used for parametric data, and the Kruskal‒Wallis test was used for non-parametric data. LEfSe analysis was performed to determine OTU biomarkers in each group based on previously described methods^42,43^. In brief, significant differences in relative abundance between each treatment were determined using two-tailed non-parametric Kruskal‒Wallis and unpaired Wilcoxon tests. LDA was performed to estimate the effect size for significantly different taxa. Only gut bacteria with a *p*­ value ≤ 0.05 and LDA scores higher than two were considered significantly different. Correlation analyses between SCFA and bacteria at the family and genus levels were performed using MetScape3 correlation calculator v1.01^44^ and visualized as a heatmap on ImageGP^43^.

### Supplementary Information

Supplementary Figure S1.

## Data Availability

The raw 16 s rRNA amplicon sequences used in this study have been deposited in the NCBI shorts read archive (SRA) under the Bio Project Accession Number PRJNA906335.
