# Supplementary material for: Synergistic activity of Limosilactobacillus reuteri KUB-AC5 and water-based plants against Salmonella challenge in a human in vitro gut model
Source: Sci Rep. 2024 Feb 27;14:4730. doi: 10.1038/s41598-024-53912-5 (PMC10899581; doi:10.1038/s41598-024-53912-5)
Supplement: Supplementary file 1 — Supplementary Figure S1. [file 41598_2024_53912_MOESM1_ESM.docx]

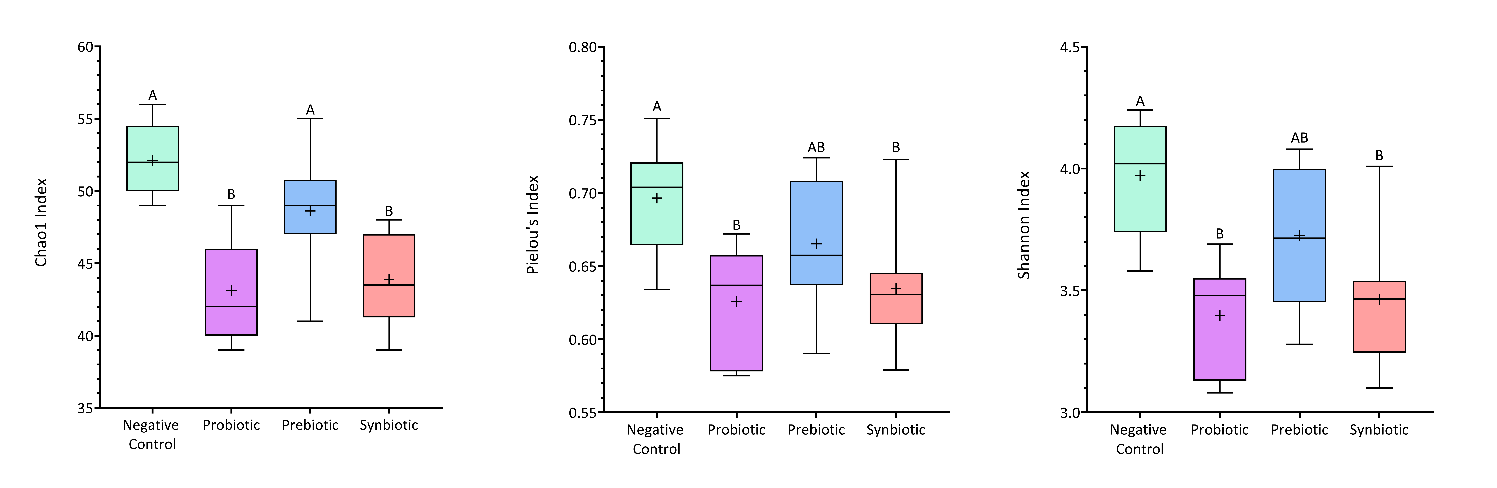
Figure S1 The alpha diversity of gut microbiota in each treatment. The horizontal line represents the median value, while the ‘+’ represents the mean value. Different letters indicated significant difference of concentration numbers between the three treatments (*p* < 0.05).
